# Metal block augmentation for bone defects of the medial tibia during primary total knee arthroplasty

**DOI:** 10.1186/1749-799X-8-36

**Published:** 2013-10-20

**Authors:** Sachiyuki Tsukada, Motohiro Wakui, Munenori Matsueda

**Affiliations:** 1 Department of Orthopaedic Surgery, Nekoyama Miyao Hospital, 14-7 Konan, Chuo-ku, Niigata 950-1151, Japan; 2 Department of Orthopaedic Surgery, Niigata Central Hospital, Niigata Japan

**Keywords:** Total knee arthroplasty, Bone deficiency, Metal, Augmentation, Stem, Midterm result

## Abstract

**Background:**

Stable and well-aligned placement of tibial components during primary total knee arthroplasty is challenging in patients with bone defects. Although rectangular block-shaped augmentations are widely used to reduce the shearing force between the tibial tray and bone compared with wedge-shaped augmentations, the clinical result remains unclear. This study aimed to evaluate the outcome of primary total knee arthroplasty with metal block augmentation.

**Methods:**

We retrospectively reviewed the 3- to 6-year follow-up results of 33 knees that underwent total knee arthroplasty with metal block augmentation (metal-augmented group) for bone defects of the medial tibia and 132 varus knees without bone defects as the control group. All surgeries were performed using posterior-stabilized cemented prostheses in both groups. Cemented stems were routinely augmented when the metal block was used.

**Results:**

There were no differences in implant survival rates (100% in metal-augmented and 99.2% in control) or knee function scores (82 points in metal-augmented and 84 points in control) between the two groups at the final follow-up examination (*P* = 0.60 and *P* = 0.09, respectively). No subsidence or loosening of the tibial tray was observed. Of 33 metal-augmented total knee arthroplasties, a nonprogressive radiolucent line beneath the metal was detected in 10 knees (30.3%), and rounding of the medial edge of the tibia was observed in 17 knees (51.5%).

**Conclusions:**

The clinical results of total knee arthroplasty with metal augmentation were not inferior to those in patients without bone defects. However, radiolucent lines were observed in 30.3%.

## Background

Peripheral bone defects of the medial tibia are frequently encountered in primary total knee arthroplasty (TKA) for varus knees [1]. Stable placement of components is difficult in these cases and thus presents technical challenges for the surgeon. Countermeasures for bone defects include increased bone resection, lateralizing of the tibial component, cement filling, bone grafting, metal augmentation, and the use of custom-made prostheses [2].

Metal augmentation is currently one of the most common countermeasures for bone defects [2]. Although wedge-shaped augmentation was previously preferred, this trend regarding the shape of metal augmentation changed to the rectangular block-shaped augmentation following a report by Fehring et al., which revealed that metal block augmentation could directly transmit torsional loads as a result of geometric interlock and could reduce cement mantle strains between the tray and tibial plateau [3]. However, to our knowledge, there has been only one clinical report of metal block augmentation during TKA [4], and that study had a major limitation of not including a control group. Although a high rate of radiolucent lines beneath the metal wedge has been reported [1,5,6], the rate of radiolucent lines beneath block-shaped augmentation has been unclear.

We evaluated clinical and radiographic outcomes of primary TKA combined with metal augmentation for bone defects and compared the clinical outcomes with those of standard TKA without bone defects. In addition, we investigated the frequency and risk factors for the development of radiolucent lines. We hypothesized that (1) clinical results were similar for TKA with and without metal augmentation and that (2) the radiolucent line beneath the tibial tray was not rare in metal-augmented TKA.

## Methods

This retrospective study was conducted with approval from the ethics committee of Nekoyama Miyao Hospital. We reviewed the clinical charts and radiographic reports of patients who underwent primary TKA between October 2006 and March 2009. The inclusion criterion for this study was patients who underwent primary TKA with metal block augmentation for medial tibial bone defects. Patients who could not be followed up for 3 years were excluded. TKAs for varus knee without bone defects were extracted and assigned serial numbers. Using simple random sampling with a random number table, we extracted four times the number of cases of metal-augmented TKAs and designated them as the control group.

For primary TKA during the study period, we selected a posterior-stabilized cemented TKA prosthesis (Scorpio nonrestrictive geometry, Stryker Orthopaedics, Mahwah, NJ, USA). In cases where tibial bone defects were expected, we prepared modular prostheses (Scorpio total stabilizer, Stryker Orthopaedics). The decision to select the modular prosthesis was finally confirmed by intraoperative findings. We performed metal augmentation when the bone defect after bone cutting comprised an area of more than 60% of a single condyle to a depth of over 5 mm; when the defect was smaller than this, cement filling and/or increased tibial bone resection was performed. During the study period, we did not use the bone grafting technique for peripheral bone defects of the medial tibia.

The Scorpio total stabilizer system has options for metal augmentation of the proximal tibia of a (1) half 5-mm block, (2) half 10-mm block, (3) full 10-mm block, and (4) 5° full wedge. Moreover, to improve stability of the tibial component, a cemented or press-fit stem could be selected. The stem can transfer loads to the diaphyseal segment of the bone. Stem augmentation is considered to provide correct component positioning, enhance fixation, and decrease stress at the bone-implant interface [7]. When needed, the offset option could be used for the stem. Although a standard cruciate-retaining or posterior-stabilized prosthesis could be selected in this system, the constrained-type prosthesis could be selected when an acceptable soft tissue balance was not achieved.

### Surgical technique

Surgeries were performed under dual lumbar and epidural anesthesia using a pneumatic tourniquet. All the arthroplasties were performed through the subvastus approach. Measured resection technique was used for bone cutting.

Before measuring the tibial bone defect, the osteophytes on the tibia were thoroughly excised. The thorough removal of tibial osteophytes often avoided the use of metal augmentation in cases in which metal augmentation was planned for tibial bone defects. Tibial osteotomy was performed perpendicular to the tibial axis in both the coronal and sagittal planes with an extramedullary rod. The amount of bone resection of the proximal tibia was determined by the thickness of the implant from the lateral tibial plateau. In the Scorpio total stabilizer system, the options for the metal block size were 5 or 10 mm. When the defect size was over 15 mm, the double-block technique described by Baek and Choi was performed [8]. A cemented stem was routinely added when metal augmentation was used. We used a 40-mm cemented stem as our first choice; however, an 80-mm cemented stem was used when the surgeon considered it adequate because of the tibial bone quality. A line connecting the medial border of the tibial tubercle with the insertion of the posterior cruciate ligament was used as a guide for the rotational position of the tibia.

Femoral osteotomy was performed using an intramedullary rod. The distal femoral osteotomy was aimed to be performed in valgus angulation equal to the angle between the anatomical and functional axes of the femur and in external rotation parallel to the epicondylar axis.

The target soft tissue balance was no varus-valgus imbalance and equal soft tissue tension in 0° extension and 90° flexion. However, complete soft tissue balance could not be easily obtained in cases of severe bone defect. Our target soft tissue tension in the 0° extension was that the soft tissue allowed full extension and avoided a recurvate knee. With regard to the varus-vulgus balance, a slightly tight medial soft tissue tension was accepted.

Patella resurfacing was performed selectively. The patella was resurfaced in patients with pain that was considered to be generated from the patellofemoral joint, with a positive patellar compression test or with severely damaged patellar cartilage.

Postoperative care was identical for patients who underwent TKA with or without metal augmentation. Weight-bearing and walking exercises were initiated 1 day after surgery. Range of motion exercises were performed without setting restrictions.

### Clinical and radiographic outcomes

We collected data regarding the following background characteristics of patients with metal augmentation and of the control patients: age, sex, height, weight, body mass index, preoperative knee function score, preoperative diagnosis, and postoperative follow-up period. For clinical results, we used the knee function score at the final follow-up examination and the implant survival rate with revision surgery (regardless of the reason) as the endpoint. Knee joint function was evaluated using the Japanese Orthopedic Association Knee Score [9,10]. This score assesses knee joint function by pain, walking ability, range of motion, and joint swelling with a perfect score of 100. The scoring system considered the Japanese lifestyle (e.g., whether a person can kneel with the tops of the feet flat on the floor and sitting on the soles) and had been proven to be significantly correlated with the Short Form 36 Health Survey Scale [10].

We grouped the preoperative tibial bone defects into peripheral and central defects according to the definitions of Dorr et al. [11]: peripheral defects offer no peripheral support for the tibial component, whereas central defects have an intact bony rim that supports the tibial component.

We categorized and assessed the radiolucent lines according to the definition by Smith et al.: nonprogressive and progressive [12]. In addition, we evaluated the following factors to determine whether they influenced the lucent line: age, sex, body mass index, and femorotibial angle.

### Statistical analysis

To compare patient background characteristics, we used the Mann–Whitney *U* and chi-square tests for continuous variables and nominal scales, respectively. In addition, we used the Kaplan-Meier method to compare implant survival rates and the log-rank test to compare the differences in survival rates between the metal augmentation and control groups. *P* < 0.05 was considered statistically significant.

## Results

From October 2006 to March 2009, primary TKA was performed on 690 joints at our institute, and we performed metal block augmentation for bone defects of the medial tibia on 34 knees (4.9%). Posterior-stabilized cemented prosthesis was used in all of 690 knees.

Thirty-three knees of 27 patients met our inclusion criterion. One knee was excluded because the follow-up period was under 3 years. As control group, 132 varus knees without bone defects were included. The demographic data of the patients are listed in Table 1. There were no significant differences between the two groups with regard to age, height, weight, body mass index, and preoperative diagnosis. However, the preoperative femorotibial angle was larger in the metal augmentation group, and the preoperative knee function score was lower in the metal augmentation group.

**Figure 1:**
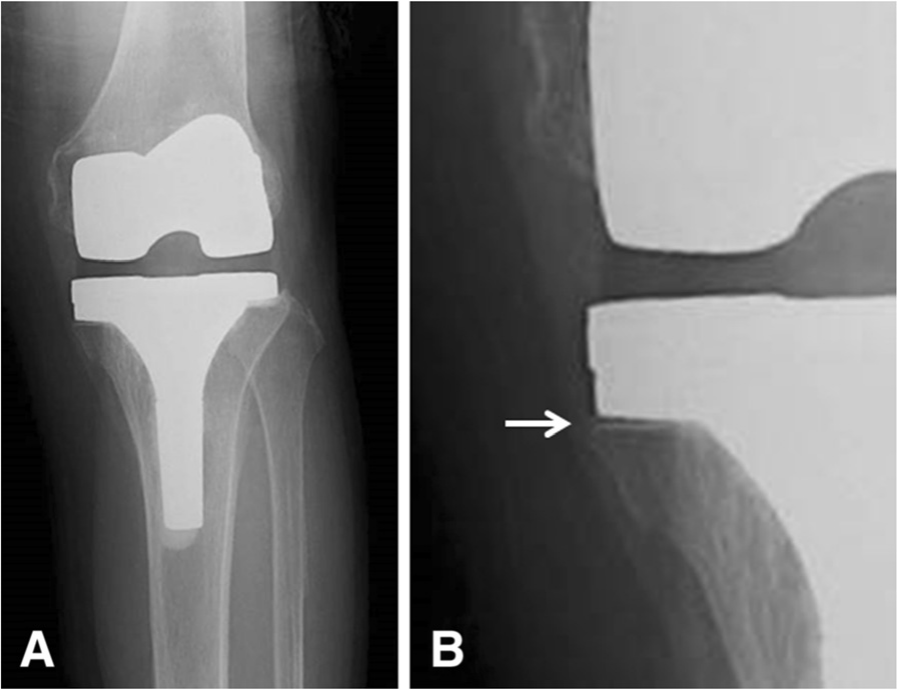
**One-year postoperative anteroposterior radiographs revealing the radiolucent line beneath the metal augmentation (arrow) in an 85-year-old woman. (A)** Overall view of radiograph. **(B)** Magnification of the medial tibia.

**Table 1:** Patient demographic and baseline clinical characteristics

	**TKA with metal augmentation (*n *= 33)**	**TKA without bone defect (*n *= 132)**	***P *value**
Age (years)	75 (58–88)	74 (50–85)	0.42^a^
Sex (F/M)	27/6	108/24	0.51^b^
Height (cm)	150 (135–172)	151 (133–176)	0.45^a^
Weight (kg)	52 (40–79)	54 (30–92)	0.10^a^
Body mass index (kg/m^2^)	23.7 (18.2–32.9)	23.7 (14.3–41.4)	0.19^a^
Preoperative diagnosis (OA/RA/other)	28/3/2	123/6/3	0.29^b^
Preoperative knee score (points)	50 (30–70)	55 (40–75)	0.01^a^
Follow-up period (years)	4.0 (3.0–6.5)	4.1 (3.0–6.8)	0.12^a^
Preoperative femorotibial angle (deg)	193 (182–205)	184 (171–192)	0.001^a^

Results are expressed as median (range) unless stated otherwise. TKA, total knee arthroplasty; OA, osteoarthritis; RA, rheumatoid arthritis. ^a^Mann-Whitney *U* test; ^b^*χ*^2^ test.

The metal half 5-mm block was used in 7 knees, and the half 10-mm block was used in 25 knees. The double block (10 mm + 5 mm) was required for one knee. Cemented 40-mm stem augmentation was used in 31 knees, and cemented 80-mm stem augmentation was used in two knees. Neither uncemented stems nor offset stems were used.

### Clinical outcomes

The median final knee function scores were 82 (range, 65–95) in the metal-augmented group and 84 (range, 45–100) in the control group (*P* = 0.09, Mann–Whitney *U* test). We observed the following postoperative complications: one skin necrosis case and one transient peroneal nerve palsy case in the metal-augmented group and one deep infection case in the control group. None of the patients with metal augmentation underwent additional surgery, whereas one implant was revised in a patient from the control group because of deep infection that developed 0.1 year after surgery. Survival rates were 100% and 99.2% (131/132 knees) for the metal-augmented and control groups, respectively (*P* = 0.60, log-rank test).

### Radiographic outcomes

Metal augmentation was performed for the 32 knees with peripheral defects and one knee with a central defect according to the Dorr classification [11]. No aseptic loosening of the tibial tray was detected at the latest follow-up in any of the cases.

On the anteroposterior radiographs, a nonprogressive radiolucent line beneath the metal wedge was observed in 10 of 33 knees (30.3%) in the metal-augmented group (Figure 1). All radiolucent lines on anteroposterior radiographs were nonprogressive according to the Smith classification [12].

**Figure 2:**
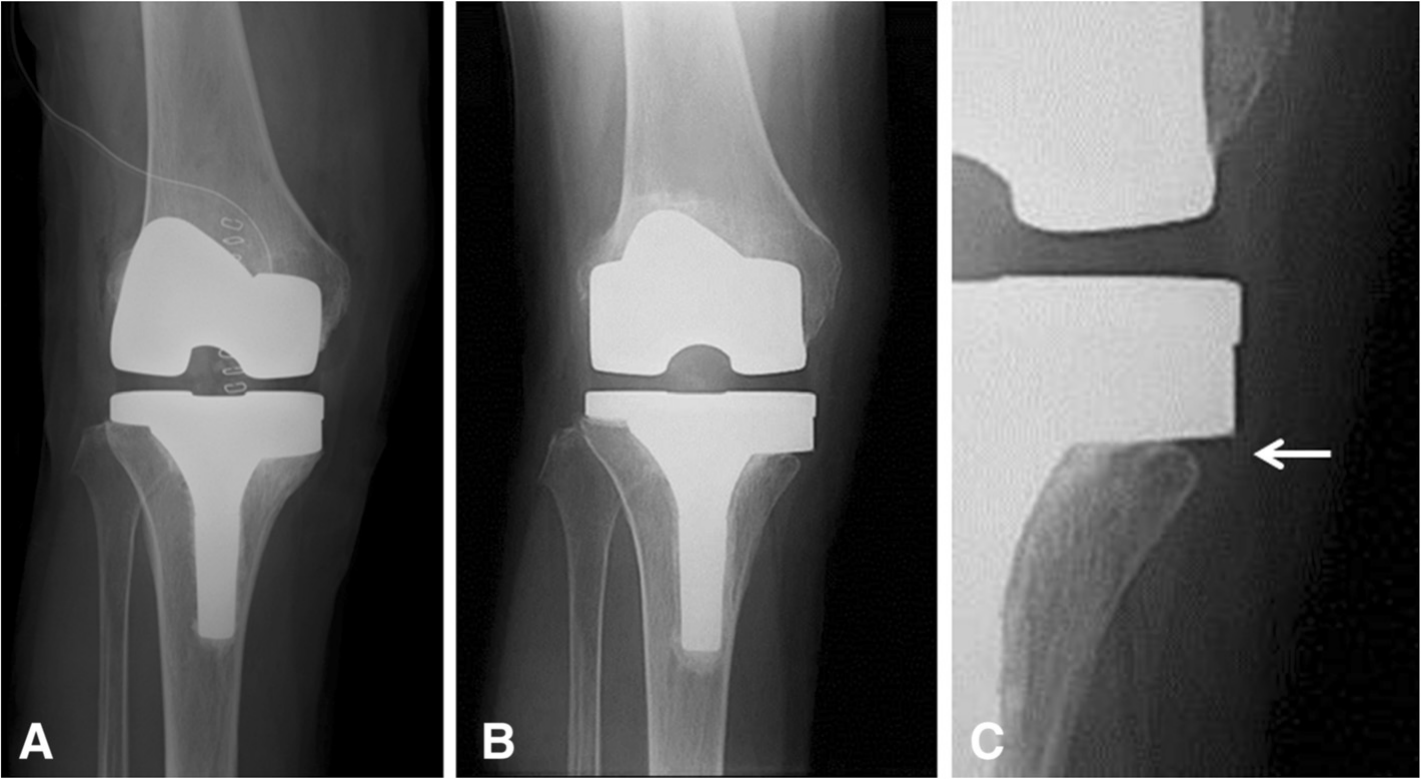
**Metal block augmentation was performed for a peripheral defect of the medial tibia in a 72-year-old woman. (A)** Immediate postoperative radiograph. **(B, C)** Radiographs recorded 5 years after surgery with rounding at the medial edge of the tibia (arrow).

On the lateral radiographs, the radiolucent lines were present in 7 of 33 knees (21.2%) in the metal-augmented group: in both zones 1 and 2 in 3 knees, only in zone 1 in 3 knees, and only in zone 2 in 1 knee. All the radiolucent lines on lateral radiographs were nonprogressive. The presence of radiolucent lines was not correlated with patient age, height, weight, body mass index, sex, side, or femorotibial angle (Table 2). Rounding of the medial edge of the proximal tibia (Figure 2) was observed in 17 of 33 (51.5%) metal-augmented knees.

**Table 2:** Patient demographic and baseline clinical characteristics associated with and without radiolucent line beneath the augmented metal block

	**TKA with radiolucent line (*n *= 10)**	**TKA without radiolucent line (*n *= 23)**	***P *value**
Age (years)	74 (67–85)	74 (58–88)	0.74^a^
Sex (F/M)	9/1	18/5	0.42^b^
Height (cm)	146 (136–153)	151 (135–172)	0.14^a^
Weight (kg)	52 (43–60)	56 (40–79)	0.37^a^
Body mass index (kg/m^2^)	23.7 (21.3–31.5)	23.7 (18.2–32.9)	0.67^a^
Preoperative diagnosis (OA/RA/other)	9/1/0	19/2/2	0.63^b^
Preoperative knee score (points)	45 (30–65)	50 (30–70)	0.14^a^
Postoperative knee score (points)	80 (65–90)	80 (65–95)	0.38^a^
Postoperative femorotibial angle (deg)	173 (170–176)	172 (167–176)	0.19^a^

Results are expressed as median (range) unless stated otherwise. TKA, total knee arthroplasty; OA, osteoarthritis; RA, rheumatoid arthritis. ^a^Mann-Whitney *U* test; ^b^*χ*^2^ test.

## Discussion

The most important finding of the study was that the 3- to 6-year follow-up results of TKA with metal block augmentation for the medial tibia were not inferior to those of TKA for varus knee without bone defects in terms of knee scores and survival rates. The use of metal augmentation for bone defects is widely supported [2]. The advantages of modular metal augmentation are extensive modularity, quick and easy use, and wide availability [7]. However, the high rate of radiolucent lines just beneath the metal has often been pointed out as a shortcoming [1,4-6]. Pagnano et al. reported radiolucent lines between wedge-type augmentation cement and bone in 13 of 24 knees with an average radiographic follow-up period of 4.8 years [6]. Brand et al. reported that 6 of 22 knees had radiolucent lines beneath the metal wedge at an average follow-up of 3.1 years [5]. Although the definitive reason for the radiolucent line is unclear, it is hypothesized to be due to insufficient cementing, thermal necrosis caused by heat from the cement, blood or tissue debris, or micromotion of components [13]. Several biomechanical studies have revealed that the rectangular block is superior to the wedge [3,14]. Block-shaped augmentation is expected to directly transmit torsional loads as a result of geometric interlock and to reduce cement mantle strains between the tray and the tibial plateau [3]. In our study, no loosening was observed over 3–6 years, and the clinical results were not inferior to those of standard TKA; however, radiolucent lines beneath the metal were observed in 10 of 33 knees.

We assessed the patients' characteristics in an attempt to detect reasons for the incidence of radiolucent lines. When the postoperative alignment of the leg was valgus, the stress to the medial part of the knee might decrease. Thus, the stress shielding of the medial part could be related to the incidence of radiolucent lines. However, in our study, no patient data, including the femorotibial angle, could be associated with the incidence of the radiolucent line.

Rounding of the medial edge of the tibia, which to our knowledge has not been previously reported, was frequently observed in our study. We consider that the rounding was caused by stress shielding, similar to the rounding of the medial calcar after total hip arthroplasty [15]. The use of stem augmentation might influence the rounding because the stem transfers the load from the tibial surface to the distal portion. The rounding suggested that the cemented stem was absorbing the load and that the metal block was not actually loading the bone as designed.

An important limitation of the present study is its retrospective nature. Furthermore, the follow-up period was not long enough to determine the usefulness of metal augmentation. Even with these limitations, we consider that these encouraging clinical results might be beneficial for TKA with tibial bone defects.

## Conclusion

The 3- to 6-year clinical outcome of block-shaped metal-augmented TKA was not inferior to that of standard TKA. In the radiographic results, the radiolucent line was not rare despite avoiding the wedge-shaped metal augmentation.

## Abbreviations

OA: Osteoarthritis; RA: Rheumatoid arthritis; TKA: Total knee arthroplasty.

## Competing interests

The authors declare that they have no competing interests.

## Authors’ contributions

ST conducted the retrospective study and drafted the manuscript. MW collected data and supervised the research. MM helped complete the manuscript. All authors read and approved the final manuscript.
